# Diversity of Volatile Compounds in Raw Milk with Different n-6 to n-3 Fatty Acid Ratio

**DOI:** 10.3390/ani12030252

**Published:** 2022-01-21

**Authors:** Ning Li, Guoxin Huang, Yangdong Zhang, Nan Zheng, Shengguo Zhao, Jiaqi Wang

**Affiliations:** 1 State Key Laboratory of Animal Nutrition, Institute of Animal Science, Chinese Academy of Agricultural Sciences, Beijing 100193, China; lining20211114@163.com (N.L.); huangguoxin1991@163.com (G.H.); zhengnan_1980@126.com (N.Z.); zhaoshengguo1984@163.com (S.Z.); 2 Laboratory of Quality and Safety Risk Assessment for Dairy Products of Ministry of Agriculture and Rural Affairs, Institute of Animal Sciences, Chinese Academy of Agricultural Sciences, Beijing 100193, China

**Keywords:** dairy cows, n-6/n-3 fatty acids, gas chromatography–ion mobility spectrometry, volatile compounds

## Abstract

**Simple Summary:**

In production, milk that is more beneficial to human health is obtained by adjusting the ratio of n-6 and n-3 fatty acids; however, the effect the regulation will have on the volatile substances in milk is unknown. In this study, gas chromatography–ion mobility spectrometry combined with principal component analysis was used to establish the fingerprint of volatile substances in raw milk to identify the types of volatile substances. The results show that a total of 34 target compounds were identified, and there were differences in the types and contents of volatile compounds among different treatment groups. The main reason for these differences is that lipid is degraded and aldehydes and ketones are produced in the adjusted-proportion group.

**Abstract:**

Fatty acid profiles may affect the flavor of milk. The diversity of volatile compounds in raw milk with different ratios of n-6 to n-3 fatty acids (8:1, 4:1, and 3:1) was studied. Gas chromatography–ion mobility spectroscopy (GC–IMS) is a promising technology for the accurate characterization and detection of volatile organic compounds in agricultural products, but its application in milk is rare or even unavailable. In this experiment, GC–IMS fingerprints along with principal component analysis (PCA) were used to study the flavor fingerprints of fresh milk samples with different percentages. Thirty-four typical target compounds were identified in total. A diversity of flavor compounds in raw milk with different n-6/n-3 was observed. After reduction of the proportion, the concentrations of volatile compounds, such as hexanoic acid (dimer and monomer), ethyl acetate, and 2-methylpropanoic acid (dimer and monomer) decreased, while those of 4-methyl-2-pentanone, pentanal, and acetone increased. We carried out PCA according to the signal strength of the identified volatile compounds, and the examination showed that it could precisely make a distinction among the samples in a comparative space. In conclusion, the results show that the volatile compounds are different as the proportion is different. The volatile compounds in raw milk are mainly hexanoic acid, ethyl acetate, and 2-methylpropanoic acid. After adjustment of the ratio, the flavor substances of the medium-ratio (MR) group were mainly ketones, while those of the low-ratio (LR) group were aldehydes. Therefore, in production, reducing the impact on volatile substances while adjusting the proportion of n-6 and n-3 fatty acids to obtain functional dairy products should be taken into consideration.

## 1. Introduction

Volatile substances in raw milk can affect its flavor, and different substances have different flavors. By determining the types of volatile substances in milk, researchers can adjust the flavor according to the taste of consumers, which is very important for manufacturers.

The flavor of high-quality milk should taste fragrant, sweet, and smooth, as well as have a feeling of abundance on the palate, without any peculiar smell and aftertaste [1]. Studies have shown that feeding dairy cows with diets rich in polyunsaturated fatty acids can produce more beneficial dairy products for the human body, which are soft in texture but tend to contain an oxidative flavor [2]. That is to say, the fatty acid profile of milk can affect the texture and flavor of milk.

At present, flaxseed is commonly used in production as a regulator of the fatty acid proportion of n-6 and n-3 in raw milk to obtain functional dairy products. Flaxseed has plentiful α-linolenic acid (ALA), which has greater stability than most polyunsaturated fatty acids containing four, five, and six double bonds, such as arachidonic acid (ARA), eicosapentaenoic acid (EPA), and docosahexaenoic acid (DHA) [3]. More importantly, n-3 polyunsaturated fatty acids can improve human health [4]. For example, ALA has a preventive effect on cardiovascular diseases and cancer [5,6], while DHA and EPA can produce resolvins, protectins, and maresins, thereby inhibiting inflammatory reactions [7]. On the contrary, n-6 fatty acids, such as ARA, can form prostaglandins and leukotrienes to promote inflammation; therefore, maintaining an appropriate proportion is important for human health [8], which is also proposed as a biomarker for coronary artery disease risk [9]. The World Health Organization as well as the Food and Agriculture Organization recommended the proportion in the diet to be approximately four to one [10], and Simopoulos has suggested that a low n-6 and n-3 ratio is more ideal for reducing the risk of many chronic diseases [11]. Riediger et al. (2009) reported that lowering the ratio can reduce cardiovascular and metabolic risks as well [12]. A low proportion can reduce proinflammatory cytokines and atherosclerosis, with overall anti-inflammatory effects [13,14,15].

Gas chromatography–ion migration spectrometry (GC–IMS) is a modern approach to detecting flavor. Compared with traditional analytical techniques, it has significant superiority in terms of high separation efficiency, good selectivity, and high sensitivity; most importantly, it does not require pretreatment, thus greatly simplifying the test operation [16]. It is widely used in food quality and safety [17,18,19], but the application in raw milk is rarely reported. In this research, the diversity of the volatile compounds was distinguished, and a Gallery Plot graph was formed. Earlier research rarely reported the detection of these target compounds in raw milk samples by GC–IMS. This research could provide a foundation for the classification and identification of raw milk samples with different proportions of fatty acids.

## 2. Materials and Methods

### 2.1. Materials

Raw milk samples were sampled from the Fuyou Agricultural Science and Technology Limited Company, Tianjin. The proportion in raw milk was adjusted by adding whole and ground flaxseed in the diet. The samples were divided into three groups: high ratio, medium ratio, and low ratio, with 10 samples in each group, respectively. The high-ratio group (HR group) was fed a diet without flaxseed. The medium-ratio group (MR group) was fed a total mixed rations (TMR) diet supplemented with 13.01% DM whole flaxseed. The low-ratio group (LR group) was fed a TMR diet supplemented with 13.01% DM ground flaxseed. The samples were packed in 50 mL centrifuge tubes and then stored in a −4 °C refrigerator for subsequent analysis. The ratio of n-6 to n-3 fatty acids in each sample is shown in Table 1.

### 2.2. Methods 

In this experiment, we used a FlavourSpec^®^ flavor analyzer (G.A.S, Dortmund, Germany) with a weak polar FS-SE-54-CB capillary column (15 m × 0.53 mm), for which the stationary phase was 5% phenyl and 95% dimethyl polysiloxane, connected to a GC–IMS unit to detect the volatile substances in 30 samples. First, the operator measured 5mL of milk from each sample and put 20 mL into 30 headspace bottles. The next step was incubation at 80 °C for 20 min, simultaneous with rotation at 500 rpm, and then injection 500 µL with the injection needle at 85 °C. Finally, the sample was transported to the FS-SE-54-CB capillary column with N_2_ gas (99.999% purity). The N_2_ gas also used as drift gas in IMS. The column temperature was constant at 60 °C. The analytes were driven into the ionization chamber and ionized in the positive ion mode. The 3H ionization source had an activity of 300 MBq. The generated ions were driven to a drift tube (9.8 cm length), which was operated at a constant temperature (45 °C) and voltage (0.5 kV). The process was shown in Table 2.

### 2.3. Statistical Analysis

The volatile substances represented by each point in the map were viewed and examined by vocal software. The reporter plug-in was used to directly compare the difference between the three-dimensional spectrum and the two-dimensional top view of the samples. The Gallery Plot plug-in component was applied to contrast the differences in flavors among different samples visually and quantitatively. In addition, the dynamic PCA made the sample cluster and defined the types of unknown samples rapidly. GC × IMS Library Search was used as the built-in NIST database of the application software to qualitatively analyze flavor substances.

## 3. Results

### 3.1. GC-IMS Topographic Plots of Different Raw Milk Samples

**Figure 1:**
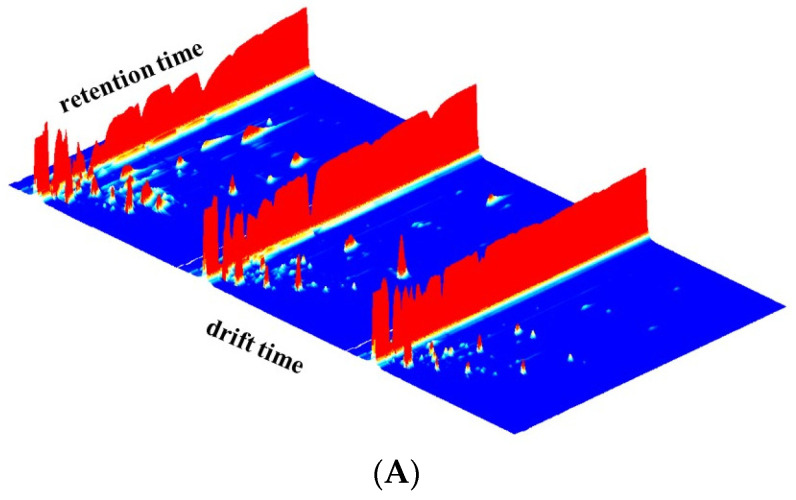

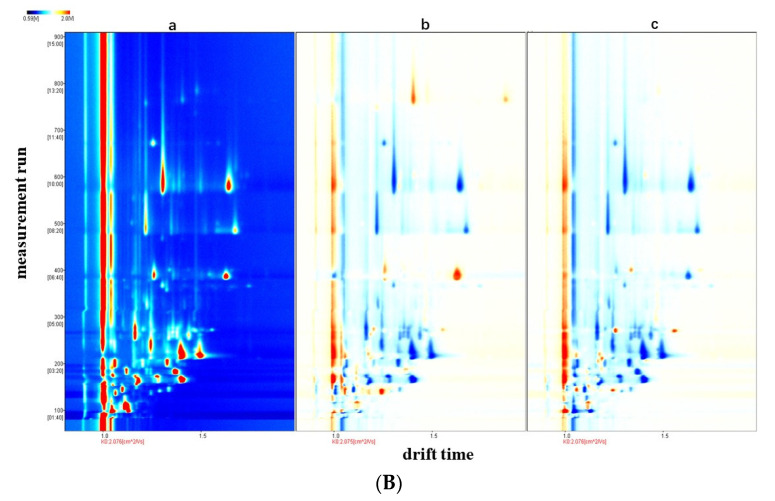
3D topography for in raw milk from different treatments. (**A**): front view. (**B**): top view. a: The ratio of n-6 to n-3 fatty acid in raw milk is 8:1. b: The ratio of n-6 to n-3 fatty acid in raw milk is 4:1. c: The ratio of n-6 to n-3 fatty acid in raw milk is 3:1.

The diversity of volatile components in different raw milk samples were analyzed by GC–IMS. The data was visualized in 3D terrain, as shown in Figure 1A. The *X*-axis is the ion migration time (i.e., drift time in milliseconds), which was used for identification; the *Y*-axis is the retention time of GC (in seconds); and the *Z*-axis represents the height of the peak, which was for quantification. The background of the whole two-dimensional map was blue. As can be seen from Figure 1A, different n-6 and n-3 ratios of volatile compounds in raw milk had different signal intensities. The HR group had the strongest milk signal: the lower the ratio, the weaker the signal.

An aerial view of the GC–IMS 3D map of raw milk samples in different ratios is shown in Figure 1B. There is a red vertical line called reaction ion peak (RIP), on the far left of the spectrum, and each dot on the right of the RIP denotes a volatile organic compound extracted from the samples. The red spots represent the higher content of flavor substances, and the white spots represent the lower content. The depth of color marks the concentration of substances; the deeper the color, the higher the content. The entire spectrum represents the whole headspace ingredients of the sample.

The difference in raw milk samples was compared by difference comparison model. The raw-milk map of the HR group as a marker was selected, and the topographic maps of other samples were removed from the reference (Figure 1B). If the concentrations of two volatile compounds were similar, the ground color subtracted was white, while red represented a higher content and blue indicated it had a lower concentration. Here, we observe that the majority of the signals appear at a retention time of around 100–700 s, and there are several different signals for the MR group (retention time was between 700 and 900 s). After reduction of the n-6 and n-3 ratio, the signal of some compounds disappeared or the signal strength decreased. On the contrary, the increase in the signal strength showed an increase in the concentration of some compounds. As shown in Figure 1B, comparison of the MR group with the HR group found that although the volatile compound concentration change in the MR group is not obvious, careful observation can be seen, and the blue part of the region increased comparable to the HR group, producing a subtle change. In the LR group, the blue part of increased more, which represented the overall volatile compound concentration of raw milk samples, decreased significantly; the LR and HR groups show a significant difference. The results indicate that the content of volatile matter in raw milk dropped with the reduction of n-6 and n-3 proportion in the HR, MR, and LR groups. The compositions of volatile compounds were also significantly different.

### 3.2. Diversities of Volatile Matter of Raw MILK samples from Different Treatments

To use the instrumental analysis to detect raw milk, fingerprint technology was used to qualitatively analyze all information, rather than just identify each volatile compound [20]. By comparing the IMS drift time and retention index of standard compounds, the detected compounds were characterized (Table 3).

Since a volatile compound may produce more than a signal or spot, monomers and dimers may be detected simultaneously; this depended on the content of volatile matter. Since the monomer and dimer belonged to the compounds with the same structure, the number was only calculated once. There are 34 target compounds identified from the GC-IMS library (Table 3), including aldehydes, esters, and ketones. Among them, there were 10 kinds of aldehydes, 9 kinds of ketones, 6 kinds of esters, and 9 kinds of other acid alcohols.

**Figure 2:**
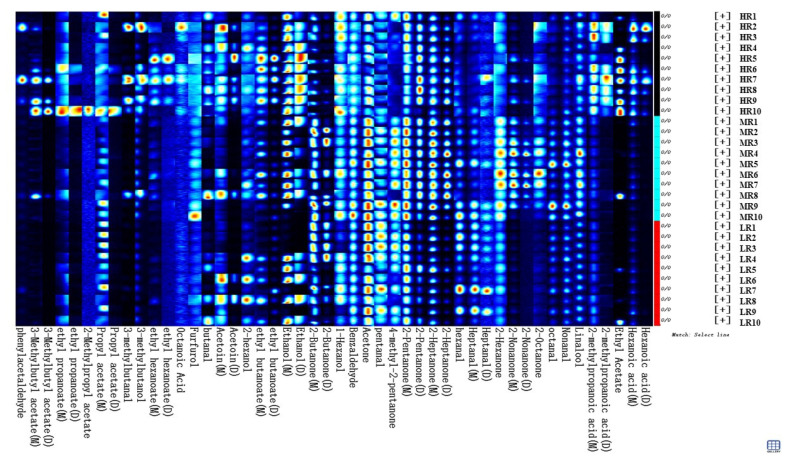
Gallery Plot of different treatments of raw milk by GC–IMS.

To compare the diversities of volatile organic compounds more comprehensively in different ratios in the raw milk sample, the Gallery Plot plug-in of the LAV software was applied to select all the peaks to be analyzed in the two-dimensional GC–IMS of raw milk samples from different treatment groups, and the graph was automatically generated. As shown in Figure 2, the left Y-axis was the sample name and the X-axis was the qualitative name of 34 the substances.

It can be seen from the preliminary comparison of fingerprints that there were differences in the composition of volatile matters among the samples. The composition of volatile substances in the three groups of raw milk samples was uneven, and the concentrations and types of some volatile substances were different. After reduction of the proportion of n-6 and n-3 fatty acids in raw milk, the types of volatile substances decreased accordingly, such as hexanoic acid (dimer, Figure 2, first column from right to left), hexanoic acid (monomer, Figure 2, from right to left of column 2), ethyl acetate (Figure 2, from right to left of column 3), 2-methylpropanoic acid (dimer, Figure 2, from right to left of column 4) and 2-methylpropanoic acid (monomer, Figure 2, from right to left of column 5). Other volatile substances were present in the HR group samples, but the contents of the MR and LR groups were low or almost none. 

On the other hand, as shown in Figure 2, the raw milk of the MR and LR groups with lower n-6 and n-3 fatty acid ratios contained more of the volatile compounds, such as 4-methyl-2-pentnone (column 20 from right to left in Figure 2), pentanal (column 21 from right to left in Figure 2) and acetone (column 22 from right to left in Figure 2). In addition, the types of volatile substances in the HR group were much richer than those in the MR group and the LR group, while the compositions of volatile substances between the MR group and the LR group were quite different. For example, the MR group was abundant in 2-nonanone (dimer, column 10 from right to left in Figure 2), 2-nonanone (monomer, column 11 from right to left in Figure 2), and 2-hexanone (column 12 from right to left in Figure 2). The LR group was abundant in heptanal (dimer, column 13 from right to left in Figure 2), heptanal (monomer, column 14 from right to left in Figure 2), and hexanal (column 15 from right to left in Figure 2), which clearly distinguished the MR group and the LR group. Fingerprints can reflect these contents more clearly than two-dimensional fingerprints can. In short, the raw milk with n-6 and n-3 fatty acid ratio of 8:1 mainly consisted of acids and esters, and the LR group had an abundance of hexanal.

### 3.3. Identification of Differences among All Samples by Principal Component Analysis(PCA)

**Figure 3:**
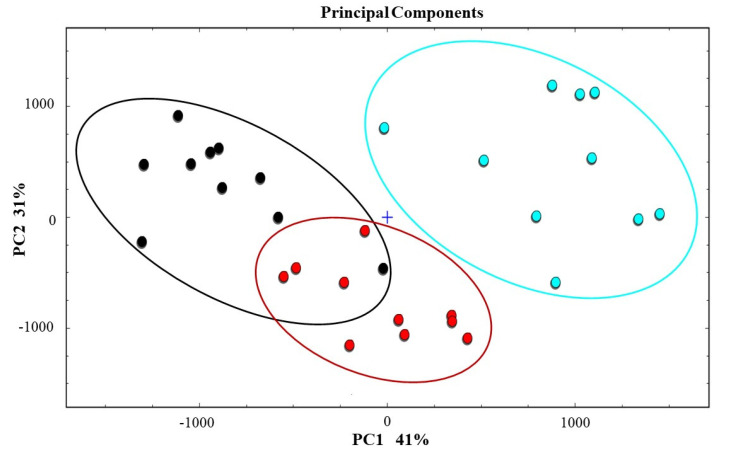
In the PCA diagram, the black points are the HR group samples (HR1-HR10), the blue points are the MR samples (MR1-MR10), and the red points are the LR samples (LR1-LR10).

In order to further explore the changes in volatile substances in milk after adding flaxseed, the PCA result is shown in Figure 3. It describes the accumulative variance contribution rates of 41% and 31%, and provides the visualization of data. These components were considered to display similarities among raw milk samples with different n-6/n-3 fatty acid ratios. 

The PCA results clearly show that raw milk samples with different proportions can be well classified in the distribution map. The MR group raw samples could be well classified by the positive value range of PC1, whereas according to the negative value of PC1, the HR group raw samples could be well classified. Combined with the score of PC2, raw milk of different groups could be separated.

As can be seen from Figure 3, the HR group samples were gathered in the left part of the PCA diagram, the LR group samples were distributed in the middle part of the diagram, and the MR group samples were distributed in the right part of the diagram. Because the compositions of volatile substances in the HR and LR groups were similar, the distribution of volatile substances in the PCA diagram was relatively close, while the composition of volatile substances in the MR group was uneven, and its distribution in the PCA diagram was more disperse. However, this did not affect the experimental results. The test demonstrated that the fingerprints of characteristic volatiles in raw milk with different ratios were successfully obtained by GC–IMS. Overall, the GC–IMS data had available information, and this technology could be a useful access to differentiating the raw milk samples.

## 4. Discussion

Some volatile compounds (aldehydes, ketones, esters, and alcohols), which are the main sources of milk flavor, were determined in our work by GC–IMS. There were 10 kinds of aldehydes, 9 kinds of ketones, 6 kinds of esters, and 9 kinds of others identified. The signals observed in each group were different because the regulation of n-6 and n-3 fatty acid ratio affected the volatile profiles of milk. The HR group samples have more intense signals and contain more diverse volatile compounds than those both in MR and LR groups.

In our study, the volatile compounds in raw milk are mainly hexanoic acid, ethyl acetate, and 2-methylpropanoic acid. According to an early report, the main specific volatile compounds of raw bovine milk are 2-butanone, 2-heptanone, ethyl caproate, heptanal, hexanal, nonanal, octanal, and pentanal [21]. This report is not consistent with our research results, probably because of the difference in the diets of the experimental dairy cows, which caused the diversity in the volatile compounds in the raw milk. This study determined the volatile compounds in cows’ milk by using headspace GC–MS, while we used GC–IMS; this is also a main reason for the diversity. Yao et al. analyzed the volatile flavor compounds in smoked chicken thighs by GC–IMS and GC–MS and found that different methods were used to obtain different flavor substances [22]. Under the synergistic effect of the two analytical methods, more comprehensive information on the flavor components of smoked chicken thighs prepared with three different smoked materials was obtained. Xiao et al. used both GC–IMS and GC–MS to characterize the volatile compounds of different sorghum cultivars and found the result of GC-IMS showed that 26 volatile compounds but not in the results from GC-MS detection [23], indicating the advantage of the methodology combination for a better understanding of the impact of cultivars and cooking on volatile characteristics of the sorghums.

Raw milk samples without fatty-acid-ratio adjustment had a more special flavor and higher volatile-compound content in our research, mainly hexanoic acid (dimer and monomer), ethyl acetate, and 2-methylpropanoic acid (dimer and monomer). Whether monomer or dimer, their contents changed identically. Brauss et al. used a model yogurt system to study and found that altering the fat content affected flavor release, which was consistent with the results of our research [24]. The raw milk without flaxseed contains more esters, because the content of acids and alcohols in HR group were comparatively high. The ethyl acetate can be synthesized via alcoholysis reaction or chemical esterification of fatty acids and alcohols [25], and it could contribute the sweet fruit flavor to raw milk, which is desirable and characteristic. Gallegos et al. used IMS for identification of some volatile metabolites from different types of goat cheese samples and found that two types of cheeses elaborated with raw goat milk were abundant in ethyl acetate, which has a fruity and pineapple flavor [26]. 

Food in the mouth undergoes mastication, salivation, bolus formation, and finally swallowing. Each step can have a significant influence on flavor release and flavor perception [27]. After addition of flaxseed to adjust the ratio, the flavor substances of the MR group were mainly ketones, while the LR group were aldehydes. They are derived from the degradation of fatty acids. Generally, aldehydes are formed from amino acids, either by enzyme-catalyzed transacylation to intermediate imines, which are then decarboxylated, or by Strecker degradation [26]. However, aldehydes also exist in milk due to lipid oxidation. These aldehydes (hexanal, heptanal, etc.) having lower threshold contributed more to milk flavor in the LR group. This also reflected to a certain extent the changes in fatty acid components in raw milk after the ratio was reduced. Chung et al. used mackerel rich in n-3 fatty acids as subjects, and they found that hexanal is a degradation product of linoleic acid [28]. In our experiment, LR group had hexanal, which is an index for evaluating the oxidation degree of n-6 fatty acid lipids. Usually, it is an intermediate product of fatty acid degradation and can be rapidly oxidized to acid by microbial activity. However, the high level of hexanal may cause off-flavors. The MR group was abundant in 2-nonanone in our study, and this compound was probably formed by oxidative decarboxylation of FA. In addition, Gallegos et al. found an increase of 2-nonanone through the time in the making of ripened cheeses [26] and it might be formed by enzymatic oxidative decarboxylation of FA by the lactic acid bacteria.

PCA is a technique for simplifying data sets that aims to use dimensional reduction to transform multi-indicators into a few comprehensive indicators [29]. The samples of the HR and LR group were closer to each other, which indicated the volatile compound content of the LR group was closer to that of the HR group. This may be because in the LR group aldehydes formed by lipid oxidation were unstable and could be rapidly oxidized into acids. It was also possible that the GC–IMS technology did not provide a comprehensive detection of volatile substances in milk. Therefore, it was necessary to establish a database of volatile substances in conjunction with other detection techniques.

## 5. Conclusions

In sum, a simple, precise, and authentic analytical approach was established to assess the characteristic volatile matters of raw milk samples by GC–IMS as well as PCA, which demanded minimal sample pretreatment procedures and reduced analysis time. PCA also revealed that the different group samples were clearly distinguishable. A total of 34 compounds from topographic plots were differentiated in raw milk samples with different fatty acid proportions in this research. Reducing the fatty-acid ratio influenced the volatile substances in raw milk, and there were three volatile substance contents that decreased or even disappeared. These differences in raw milk could be clearly observed from the samples in the low-proportion-fatty-acid (MR and LR) groups. On the other hand, we can subsequently use different testing methods to analyze volatile substances in raw milk to enrich volatile-compound information. Finally, in the production, reducing the impact on volatile substances while adjusting the proportion of n-6 and n-3 fatty acids to obtain the functional dairy products should be taken into consideration.

## Figures and Tables

**Table 1:** The ratio of n-6 to n-3 fatty acids in each group of samples.

Treatments	n-6/n-3	Treatments	n-6/n-3	Treatments	n-6/n-3
HR1	8.604	MR1	3.722	LR1	3.091
HR2	7.908	MR2	5.237	LR2	3.111
HR3	8.005	MR3	3.575	LR3	4.509
HR4	8.457	MR4	4.599	LR4	3.133
HR5	7.961	MR5	5.189	LR5	3.037
HR6	8.320	MR6	4.814	LR6	3.030
HR7	7.924	MR7	4.215	LR7	3.113
HR8	8.523	MR8	4.260	LR8	2.880
HR9	8.670	MR9	4.426	LR9	3.365
HR10	7.991	MR10	4.502	LR10	3.233
Average	8.236	Average	4.454	Average	3.250

HR = high-ratio; MR = medium-ratio; LR = low-ratio.

**Table 2:** The information of velocity of drifting gas (E1), carrier gas (E2), and the recording (R) of the beginning and ending.

Time	E1	E2	R
00:00	150 mL/min	2 mL/min	Rec
02:00	150 mL/min	2 mL/min	-
10:00	150 mL/min	15 mL/min	-
20:00	150 mL/min	80 mL/min	-
25:00	150 mL/min	130 mL/min	Stop

**Table 3:** Qualitative analysis of volatile organic compounds in raw milk samples, including CAS registry number (CAS No.), molecular weight (MW), retention index (RI), retention times (RT), and relative migration time (DT).

Count	Compound	CAS No.	Formula	MW	RI	RT [sec]	DT [a.u.]	Structure
1	Nonanal	C124196	C_9_H_18_O	142.2	1104.8	784.727	1.47794	
2	2-Nonanone	C821556	C_9_H_18_O	142.2	1097.9	769.582	1.40903	Monomer
	2-Nonanone	C821556	C_9_H_18_O	142.2	1096.1	765.851	1.88047	Dimer
3	Phenylacetaldehyde	C122781	C_8_H_8_O	120.2	1049.7	672.112	1.25843	
4	ethyl hexanoate	C123660	C_8_H_16_O_2_	144.2	1012.8	605.99	1.34128	Monomer
	ethyl hexanoate	C123660	C_8_H_16_O_2_	144.2	1010.8	602.707	1.79409	Dimer
5	Octanal	C124130	C_8_H_16_O	128.2	1012.2	605.052	1.41127	
6	Hexanoic acid	C142621	C_6_H_12_O_2_	116.2	998.1	581.604	1.30985	Monomer
	Hexanoic acid	C142621	C_6_H_12_O_2_	116.2	999.9	584.418	1.64411	Dimer
7	Benzaldehyde	C100527	C_7_H_6_O	106.1	957.9	501.045	1.14803	
8	2-Heptanone	C110430	C_7_H_14_O	114.2	891.5	390.856	1.26047	Monomer
	2-Heptanone	C110430	C_7_H_14_O	114.2	889.0	387.203	1.63389	Dimer
9	3-Methylbutyl acetate	C123922	C_7_H_14_O_2_	130.2	877.8	371.375	1.30767	Monomer
	3-Methylbutyl acetate	C123922	C_7_H_14_O_2_	130.2	876.0	368.94	1.74911	Dimer
10	1-Hexanol	C111273	C_6_H_14_O	102.2	868.8	359.199	1.32572	
11	ethyl butanoate	C105544	C_6_H_12_O_2_	116.2	797.2	275.188	1.20633	Monomer
	ethyl butanoate	C105544	C_6_H_12_O_2_	116.2	795.4	273.361	1.55893	Dimer
12	Hexanal	C66251	C_6_H_12_O	100.2	793.6	271.535	1.25492	
13	2-Pentanone	C107879	C_5_H_10_O	86.1	685.8	183.262	1.12026	Monomer
	2-Pentanone	C107879	C_5_H_10_O	86.1	683.4	182.044	1.37014	Dimer
14	2-Butanone	C78933	C_4_H_8_O	72.1	584.9	138.821	1.06196	Monomer
	2-Butanone	C78933	C_4_H_8_O	72.1	586.5	139.43	1.24798	Dimer
15	Acetone	C67641	C_3_H_6_O	58.1	503.0	110.817	1.11888	
16	4-methyl-2-pentanone	C108101	C_6_H_12_O	100.2	736.9	220.398	1.1744	
17	Acetoin	C513860	C_4_H_8_O_2_	88.1	714.6	203.091	1.06014	Monomer
	Acetoin	C513860	C_4_H_8_O_2_	88.1	714.6	203.091	1.33335	Dimer
18	3-methylbutanol	C123513	C_5_H_12_O	88.1	735.4	219.246	1.5004	
19	Ethyl Acetate	C141786	C_4_H_8_O_2_	88.1	607.2	147.615	1.33495	
20	Ethanol	C64175	C_2_H_6_O	46.1	457.3	97.718	1.05001	Monomer
	Ethanol	C64175	C_2_H_6_O	46.1	465.7	100.021	1.13238	Dimer
21	3-methylbutanal	C590863	C_5_H_10_O	86.1	645.7	164.119	1.4073	
22	ethyl propanoate	C105373	C_5_H_10_O_2_	102.1	706.4	197.127	1.15019	Monomer
	ethyl propanoate	C105373	C_5_H_10_O_2_	102.1	709.1	199.046	1.45405	Dimer
23	Propyl acetate	C109604	C_5_H_10_O_2_	102.1	712.7	201.733	1.16243	Monomer
	Propyl acetate	C109604	C_5_H_10_O_2_	102.1	710.1	199.814	1.47854	Dimer
24	2-Methylpropyl acetate	C110190	C_6_H_12_O_2_	116.2	767.5	246.639	1.61544	
25	2-methylpropanoic acid	C79312	C_4_H_8_O_2_	88.1	782.4	260.457	1.16243	Monomer
	2-methylpropanoic acid	C79312	C_4_H_8_O_2_	88.1	782.4	260.457	1.37391	Dimer
26	Linalool	C78706	C_10_H_18_O	154.3	1088.7	749.932	1.22063	
27	Octanoic Acid	C124072	C_8_H_16_O_2_	144.2	1184.8	982.302	1.45182	
28	2-Octanone	C111137	C_8_H_16_O	128.2	997.6	580.767	1.33443	
29	Furfurol	C98011	C_5_H_4_O_2_	96.1	827.9	308.433	1.08207	
30	2-hexanol	C626937	C_6_H_14_O	102.2	776.7	255.096	1.28405	
31	Heptanal	C111717	C_7_H_14_O	114.2	898.3	400.876	1.33955	Monomer
	Heptanal	C111717	C_7_H_14_O	114.2	898.9	401.71	1.69995	Dimer
32	Pentanal	C110623	C_5_H_10_O	86.1	697.8	191.007	1.1889	
33	Butanal	C123728	C_4_H_8_O	72.1	590.9	141.125	1.2903	
34	2-Hexanone	C591786	C_6_H_12_O	100.2	785.5	263.444	1.18811	

## Data Availability

Not applicable.
